# Drug-Loaded Chitosan Scaffolds for Periodontal Tissue Regeneration

**DOI:** 10.3390/polym14153192

**Published:** 2022-08-05

**Authors:** Gamal Abdel Nasser Atia, Hany K. Shalaby, Mehrukh Zehravi, Mohamed Mohamady Ghobashy, Hager Abdel Nasser Attia, Zubair Ahmad, Farhat S. Khan, Abhijit Dey, Nobendu Mukerjee, Athanasios Alexiou, Md. Habibur Rahman, Joanna Klepacka, Agnieszka Najda

**Affiliations:** 1 Department of Oral Medicine, Periodontology, and Diagnosis, Faculty of Dentistry, Suez Canal University, Ismailia P.O. Box 41522, Egypt; 2 Department of Oral Medicine, Periodontology and Oral Diagnosis, Faculty of Dentistry, Suez University, Suez P.O. Box 43512, Egypt; 3 Department of Clinical Pharmacy Girls Section, Prince Sattam Bin Abdul Aziz University, Al-Kharj 11942, Saudi Arabia; 4 Radiation Research of Polymer Chemistry Department, National Center for Radiation Research and Technology (NCRRT), Atomic Energy Authority, Cairo P.O. Box 13759, Egypt; 5 Department of Molecular Biology and Chemistry, Faculty of Science, Alexandria University, Alexandria P.O. Box 21526, Egypt; 6 Unit of Bee Research and Honey Production, Faculty of Science, King Khalid University, P.O. Box 9004, Abha 61413, Saudi Arabia; 7 Biology Department, College of Arts and Sciences, Dehran Al-Junub, King Khalid University, P.O. Box 9004, Abha 61413, Saudi Arabia; 8 Department of Life Sciences, Presidency University, Kolkata 700073, India; 9 Department of Microbiology, Ramakrishna Mission Vivekananda Centenary College, Khardaha 700118, India; 10 Department of Science and Engineering, Novel Global Community Educational Foundation, Hebersham, NSW 2770, Australia; 11 Department of Global Medical Science, Wonju College of Medicine, Yonsei University, Wonju 26426, Korea; 12 Department of Commodity Science and Food Analysis, Faculty of Food Science, University of Warmia and Mazury in Olsztyn, Oczapowskiego 2, 10-719 Olsztyn, Poland; 13 Department of Vegetable and Herbal Crops, University of Life Science in Lublin, Doświadczalna Street 51A, 20-280 Lublin, Poland

**Keywords:** chitosan, periodontitis, scaffold, regeneration, tissue engineering

## Abstract

Chitosan is a natural anionic polysaccharide with a changeable architecture and an abundance of functional groups; in addition, it can be converted into various shapes and sizes, making it appropriate for a variety of applications. This article examined and summarized current developments in chitosan-based materials, with a focus on the modification of chitosan, and presented an abundance of information about the fabrication and use of chitosan-derived products in periodontal regeneration. Numerous preparation and modification techniques for enhancing chitosan performance, as well as the uses of chitosan and its metabolites, were reviewed critically and discussed in depth in this study. Chitosan-based products may be formed into different shapes and sizes, considering fibers, nanostructures, gels, membranes, and hydrogels. Various drug-loaded chitosan devices were discussed regarding periodontal regeneration.

## 1. Introduction

Periodontitis is defined as multifactorial inflammation produced primarily by the deposition of bacterial pathogens in the periodontal tissues, which gradually damages the supporting tissues of dentition [1,2]. Immunologic responses enhance inflammatory response and tooth movement, perhaps leading to teeth loss rather than treating the condition [3]. Existing periodontal therapies, including first-line therapy, surgery, and guided tissue regeneration, can lower periodontal damage and partially repair periodontal attachment, but the results are inadequate [4]. To facilitate periodontal regeneration, local medication delivery devices for the periodontal cavity are being developed [5]. Various drug carriers for local therapy have been generated for the treatment of periodontitis [6]. The actual objective of periodontal tissue engineering is to establish a place in which viable cells can proliferate [7]. One of the most critical difficulties in the administration of biologically active medicines is effective drug delivery. Nevertheless, in the complex milieu of tissue regeneration, the accessibility of biomolecules may be restricted by undesirable dissolution, adaptation, short half-lives, adverse reactions, multi-drug resistance, and nonspecific targets [8]. Due to their burst release, quick degradation, or low target specificity, biomolecules with limited bioavailability are challenging to employ in hard tissue regeneration [9]. Therefore, the development and application of drug delivery systems (DDSs) that are sustained-release, non-toxic, non-immunogenic, and biodegradable are essential for overcoming these limits. The paradigm of biomaterials, which has been widely promoted in recent times, has made great progress in the development and application of safe and effective substances [10]. Biomaterials are natural, synthetic, or semi-synthetic compounds that are intended for use in biological contexts [11]. There are effective clinical applications of biomaterials, mostly in reconstructive cosmetic procedures and orthopedic surgery, to repair the morphology and restore or increase the performance of organs and systems [12], and in the dental field [13,14]. Natural biomaterials derived from plants and animals that are mostly of polysaccharide origin (such as chitin) or protein-derived (such as collagen) have distinct advantages over the conventional synthetic polymers, including biocompatibility, processability, comprehensive availability, and exceptional biological activities [15]. Over the years, the use of biopolymers derived from nature in bioengineering, delivery of biomolecules, and other medicine and food manufacturing has demonstrated their enormous promise in biological applications [16]. Polysaccharides, a category of natural macromolecules, have high bioactivity and are often generated from crop production or crustacean shell wastes utilizing various biotechnological techniques. In terms of accessibility, CT ranks second only to cellulose, with over 10 gigatons accessible yearly [17]. Chitin existence in nature are shown in Table 1.

## 2. History and Extraction of Chitosan

**Figure 1:**
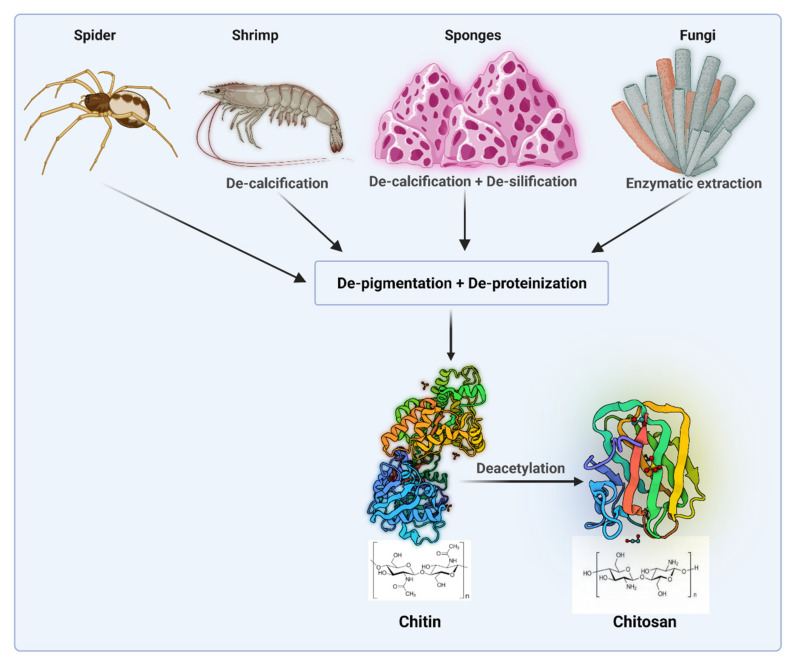
Sources and extraction of chitosan (Created with BioRender.com access on 1 February 2021).

Chitosan (Cs) is a natural polysaccharide originated from chitin via DE acetylation process. Figure 1 shows sources and extraction of chitosan.

Chitosan research has garnered a lot of interest throughout the years. Rouget discovered the deacetylation derivative of chitin in 1859, which Hoppe-Seyler subsequently termed chitosan (1894) [17]. The composition and order of such molecules will define the polymer’s physicochemical as well as its biological characteristics [18,19]. Chitosan is a fascinating biomaterial with great qualities such as biodegradability, biocompatibility, antimicrobial properties, low antigenicity, hygroscopicity, and moisturizing capabilities. CS offers a broad variety of applications regarding tissue engineering [20,21,22], wound healing [23] as additives for drug delivery [24], gene transfer], and can be formulated according to manufacturer needs [25,26,27,28]. Chitosan’s benefits and drawbacks as a local medicine delivery vehicle are shown in Table 2.

## 3. Structure of Chitosan and Its Derivatives

**Figure 2:**
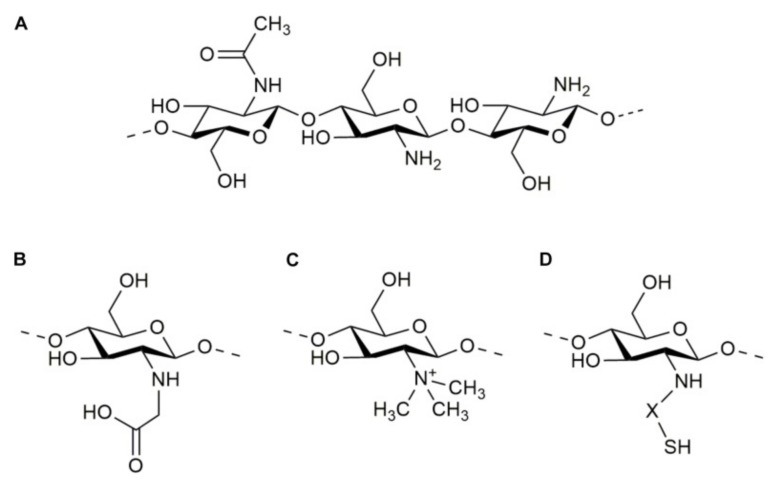
Molecular structure of chitosan (**A**) and some of its derivatives: N-carboxymethyl chitosan (**B**), N-trimethyl chitosan (**C**), and thiolated chitosan (**D**).

Chitosan units have an amino/acetamido group at C-2, a secondary hydroxyl group at C-3, and a primary hydroxyl group at C-6 in their chemical structure (Figure 2A). As a result, the improved changes implemented for this polysaccharide utilize advantage of these compounds by attaching additional molecules.

Carboxyalkylation, thio-lation, sulfation, phosphorylation, esterification, graft copolymerization, and cross-linking methods are examples of these changes. These adjustments provide the resulting goods new and distinct features [29].

### 3.1. Carboxymethyl Chitosan (CMC)

The incorporation of carboxyalkyl units into the backbone of chitosan, as carboxymethyl, was designed primarily to increase chitosan solubility. The process takes place at either the C6 hydroxyl group or the NH2 component, producing N-CMC, O-CMC, or N,O-CMC as products. Chitosan-carboxymethylchitosan blending membrane was fabricated and had desirable biocompatibility, making it a good candidate for guided periodontal regeneration [30].

### 3.2. N-Trimethyl Chitosan (TMC)

The insertion of different alkyl groups at the amino units of chitosan is known as methylation. TMC is the most prevalent byproduct of these reactions, and because of its cationic character, it is regarded as one of the powerful mucoadhesive polymers [31]. Quaternary ammonium chitosan, i.e., *N*,*N*,*N*-trimethyl chitosan, a liposome, and doxycycline (TMC-Lip-DOX NPs) inhibited biofilm formation, and showed excellent biocompatibility with human periodontal ligament fibroblasts [32].

### 3.3. Thiolated Chitosan

Thiolation is the interaction of chitosan’s main amino groups with thiol-containing coupling chemicals (thioglycolic acid, 2-iminothiolane, cysteine, and thiobutylamidine). This reagent offers excellent permeability, adhesiveness, and solubility at physiologic pH levels, as well as in situ gelling capabilities [33].

A scaffold consisting of N,*N*,*N*-trimethyl chitosan, a liposome, and doxycycline nanoparticles (TMC-Lip-DOX NPs) was developed, and showed excellent biocompatibility with human periodontal ligament fibroblasts, making it a good candidate for application in periodontal therapy and other inflammatory diseases [32].

### 3.4. Grafting Copolymerization of Chitosan

Chitosan is commonly coupled with other polymers to achieve copolymerization. Chitosan is commonly grafted with other polymers to achieve copolymerization. The graft polymer is chosen based on its biochemical, structural, or physiological characteristics, and copolymerization produces a chitosan-based compound with enhanced features [34]. For example, poly (ε-caprolactone)-poly(ethylene glycol) (PCE) copolymer was grafted with porous chitosan, and improved the orientation of collagen fibers in regenerated periodontium [35].

## 4. Biological Characteristics of Chitosan

**Figure 3:**
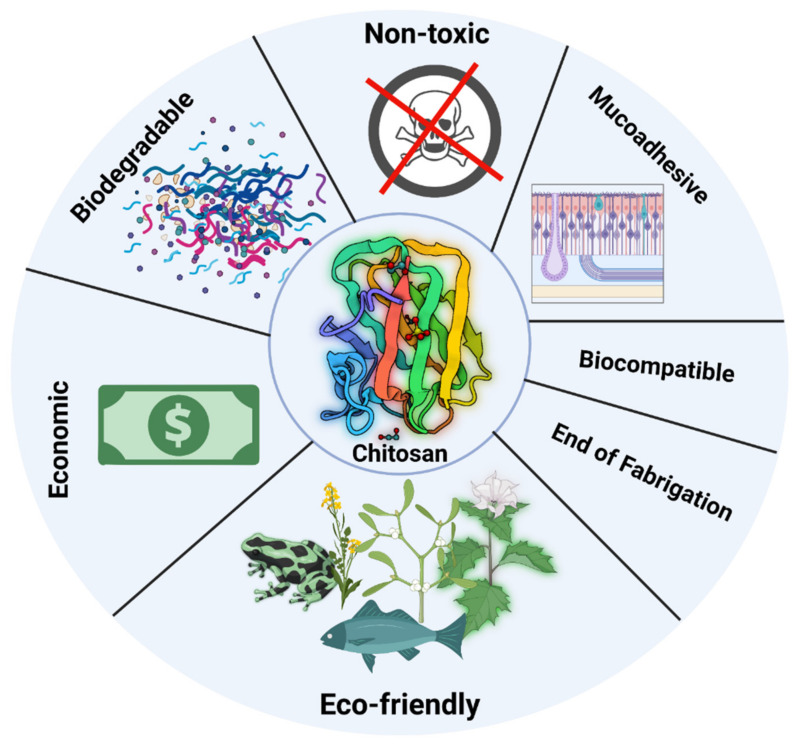
Shows multiple properties of chitosan (Created with BioRender.com).

Chitosan is a biodegradable and biocompatible. Many nations have authorized it for nutritional use as well as a wound dressing (e.g., Japan, and Finland). Some chitosan adjustments, nevertheless, may make it more or less poisonous, and any leftover reagents must be carefully removed [36,37]. Biodegradation is critical for chitosan’s various metabolic processes and material recycling in nature. Chitosan degradation in vivo was determined by its molecular weight and degree of deacetylation [38]. It has antibacterial properties against numerous fungi, bacteria, viruses, and parasites due to its abundance of amino groups. Its antibacterial action is driven by its amount, degree of deacetylation, and the test microorganism [39]. It is commonly acknowledged that chitosan has positive charges, which may enable it to interact with anionic groups from microorganism cell membranes and modify membrane permeability, causing microbial cells to decrease. The availability of natural chitosan in some species provides a crucial biological foundation for their resistance to bacteria and viruses. Chitosan also has other biological features, for example cytocompatibility, biodegradability, mucosal adhesion, and anti-inflammatory actions. With these properties, chitosan has found enormous applications [40]. Figure 3 shows multiple properties of chitosan.

## 5. Methods of Fabrication of Chitosan Products

There are various technologies for manufacturing chitosan products, according to the manufacturer’s needs. Table 3 shows various Fabrication methods of chitosan products, their application, and advantages and disadvantages.

### 5.1. Ionic Gelation Process

Since the 1990s, ionotropic gelation has been implemented in the development of polymeric biomaterials [50]. Chitosan structures encapsulating antibiotics [51], antioxidants [52] and growth mediators [53] have been acquired, exemplifying how adaptable this method is. This technique has so many advantages: it is simple, economical, and needs no complicated equipment or solvents, and takes a very short time to be completed [54]. The main disadvantage is low mechanical stability of polymeric products yielded from this process [55]. Many research groups concentrate on ways to better couple medications or genes to chitosan nanoparticles. 

Many researchers are working on chitosan and its derivative nanoparticles utilizing the ionic gelation process, which can efficiently package biomolecules, convey the targeted medicine throughout the body, and gradually deliver drugs in a regulated manner. Its derivative nanoparticles employ the ionic gelatinization, which can successfully encapsulate biological macromolecules, deliver the specific medicine to the body, and manage the drug’s gradual distribution [56]. One of the potential development directions of this innovation is the composite technology, which combines nanoparticles with other techniques to combine the benefits of many forms while avoiding the faults of a specific dosage form.

### 5.2. Solvent Evaporation Technique

The solvent evaporation method is one of the easiest methods when compared to other techniques. To create the emulsion, chitosan solution will be first added to the aqueous phase. The polymer-solvent then dissipates and precipitates, resulting in the formation of nano spheres. After adding pDNA-Tris buffer and ethanol, the mixture was shaken for 30 min with vigorous stirring. The solvent is eliminated by providing lower pressure, resulting in nanoparticles. Finally, the extract was filtered, resulting in nanoparticles [57]. Jonacir Novaes et al. used solvent evaporation to create chitosan and collagen mixes with silver nanoparticles [58]. 

### 5.3. Reverse Micellar Method

Reversible micelles are aqueous solutions of water, oil, and chemicals that are separated into water and oil microspheres by surfactant-rich barriers and are significantly more thermally stable. Surfactants are amphiphilic substances that readily establish spherical or elliptical aggregates in water or organic solvents. Traditional micelles appear in water with diminished levels of organic solvents, whereas reverse micelles form in water with high accumulation of organic solvents [59].

### 5.4. Cross-Linking

Physically cross-linked chitosan scaffolds are easy to be produced, safe and non-toxic in comparison with chemically cross-linked chitosan. The physical hydrogels involve various physical interactions such as interactions between molecules, hydrogen bonding, ionic, and hydrophobic bonds [60]. While chemical cross-linking involves the development of chemical bonds between polymer molecules and initiated through change in pH and radiation, etc. [61,62,63,64,65].

### 5.5. Self-Assembling Method

The introduction of the self-assembly approach solves the many issues, such as introduction of hazardous chemicals or use of complex construction parameters, such as high temperature or pressure [66]. Chitosan has positive charges, whereas phospholipids have negative charges. The electrostatic interactions between them resulted in the formation of nanoparticles [67]. The entire process used no organic reagents or cross-linking agents, protecting the delivered biomolecules from harmful destruction. Because chitosan nanostructures are easy to create, bulk products, and have high entrapment effectiveness, they are predicted to be widely used in clinics [68,69].

### 5.6. Sieving Method

This approach comprises the production of a 4 percent chitosan hydrogel containing the drug, followed by the addition of a cross-linking reagent, for example glutaraldehyde to generate a cross-linked chitosan hydrogel that is pushed across a sieve of a certain size to obtain the drug-loaded micro particles. The residual glutaraldehyde is eliminated by washing the resulting micro particles with 0.1 N sodium hydroxide and drying them in a 40 °C furnace [70].

### 5.7. Spray Drying Method

The basic idea of spray drying is to evaporate atomized drops with a stream of hot air. To obtain the necessary particles, an aqueous chitosan-protein solution is prepared and sprayed through a pump into a drying vessel. Proteins incorporated into chitosan micro particles using this approach include Bovine serum albumin and salmon calcitonin [71].

### 5.8. Freeze-Drying

Freeze-drying is an industrial procedure that is employed to assure the long-term durability and preservation of pharmaceutical and biomedical products [41]. Freeze-drying works by freezing the material, lowering the surrounding pressure, and applying sufficient heat to enable the substance’s frozen water to sublimate straight from the solid matrix to the gaseous state. The core components are encapsulated by freeze-drying as they homogenize in matrix solutions and subsequently co-lyophilize, resulting in unknown morphologies.

## 6. Chitosan-Derived Drug Delivery Systems

**Figure 4:**
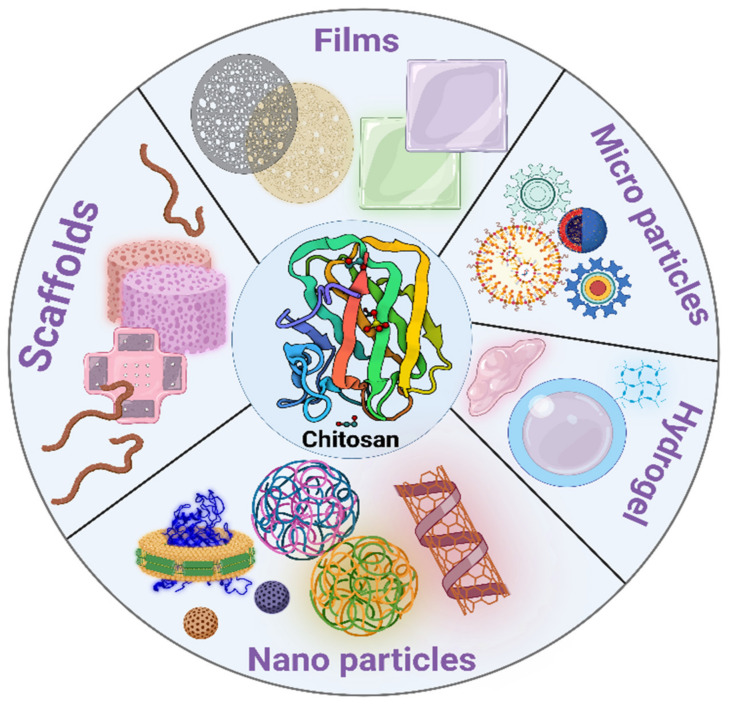
Different formulations of chitosan (Created with BioRender.com).

The physical rebuilding of chitosan and its derivatives provides a plethora of options for various applications. Chitosan-based drug carriers are a promising method for delivering therapeutic medicament doses into periodontal tissues. Table 4 shows advantages and disadvantages of different chitosan formulations Chitosan-based fibers, films, sponges, and injectable systems such as micro particles, nanoparticles, and gels are among the delivering modalities developed for sustained medicine release into the periodontal tissues [72]. Figure 4 shows different formulations of chitosan.

### 6.1. Microspheres

Chitosan microspheres are circular structures with sizes starting at few micrometers to 1000 m that are manufactured to contain bioactive molecules in a homogenous distribution in the polymer matrices using thermal and laboratory cross-linking, spray drying, solvent evaporation, and emulsion mechanisms [73]. One disadvantage of this delivery form is initial drug burst activity, which affects the sustainability and encapsulation efficiency of drug-loaded chitosan micro/nanoparticles, is being countered by covering the particles with various anionic polysaccharides, like hyaluronic acid [74]. Yadav et al. created a chitosan/alginate based microspeheric scaffold for the concurrent sustained delivery of doxycycline and ornidazole. The biocompatibility, mucoadhesiveness, and antimicrobial features of CS/Alg microspheres were proven in this study, as well as a full understanding of the polyelectrolyte complexes [75]. Microspheres have been hypothesized as possible vehicles for active material delivery into the periodontal pocket. Because the irritated periodontium is an inflamed tissue, the local delivery system should alleviate the patient’s pain and discomfort and be held in the patient’s pocket for the requisite period of time [76].

### 6.2. Nanoparticles

Regarding all kinds of vehicles made of chitosan, and because of their superior features, chitosan nanoparticles (CSNPs) have a bright potential in the treatment of oral diseases. When compared to the other delivery methods, nanoparticle systems have various advantages, including great dispensability in an aqueous medium and improved stability. Nanoparticles, due to their small size, can reach areas that conventional delivery techniques cannot, such as periodontal pockets beneath the gum line. These technologies reduce the need for re-administration while simultaneously providing homogenous dispersion of the active compounds over a lengthy period of time [77,78]. Furthermore, nanoparticles may be used as a drug carrier means to provide an optimal release of drug in the periodontium [79]. This technology consists of biodegradable bio adhesive nanoparticles encased within a humidity-responsive micro particle. Their lucrative usage in doxycycline encapsulation], silver nanoparticles [80], and tetracycline and have been confirmed. CSNPs containing minocycline, a tetracycline having anti-inflammatory and anti-catabolic properties, were tested for biomedical applications inside human gingival fibroblasts. This composition exhibited no negative effects on the behavior of human gingival fibroblasts, including cell survival, structure, and metabolic functions, and it also triggered the suppression of inflammatory cytokines [81].

### 6.3. Films

An embedded film, which is frequently used as an intrapacket vehicle, is another type of chitosan-based matrix delivery device [82]. Films produced by using solvent casting processes are classified as biodegradable or non-biodegradable based on their purpose, which is primarily the distribution of hydrophilic substances, for example chlorhexidine gluconate [83]. Biodegradable films are the recommended kind of carrier for administration in periodontal pockets due to their ease of implantation without interfering with the patient’s diet, dental hygiene routines, and everyday activities. Another benefit of films over other devices is that the form and size of these biomolecules may be tailored to the area of the periodontal defect [84]. Because of the significant volume of saliva, the lubricating environment of the buccal mucosa compromises the longer preservation of films. As a result, one of the required features for manufactured films is excellent mucoadhesiveness.

### 6.4. Gels

Gels are semisolid, resilient, soft, pliable, and high-water-content polymers that are ideal for tissue engineering and local compound/transcription factor delivery because to their great adhesiveness, cytocompatibility, and processability [85]. Hydrogels are often composed of more than 90% water and exhibit typical physical behavior such as dependability and stability, physical strength, uniformity, and processability [86]. Chitosan may be used to make two types of hydrogels: chemical gels cross-linked using multifunctional agents such as epichlorohydrin [87], glutaraldehyde [88], diethyl squarate [89], and physical gels which are formed from low energy junctions [90]. Hydrogels incorporate drug macromolecules in their polymer network, preventing disintegration and controlling long-term release [91,92]. Numerous oleogels and hydrogels have been explored for the administration of tetracycline (2.5%), metronidazole (25%), metronidazole benzoate (40%), and a combination of tetracycline (2.5%) and metronidazole benzoate (40%), with promising results. Bio adhesion or mucoadhesion is required for prolonged drug release at the area [93]. Hydrogels must have the necessary consistency, synergy, extended pharmacokinetic profile, and strong mucoadhesiveness for extended retention in the location in order to be placed into the periodontium [94]. In this context, Ozdogan et al. demonstrated that drug-loaded chitosan hydrogel had superior characteristics than chitosan base gel [95]. 

### 6.5. Fibers

Fibers, also known as thread-like implants, are reservoir-type mechanisms that are put a periodontal tissues coronally with an instrument and attached with cyanoacrylate adhesive to allow for the prolonged release of the encapsulated medicament into the pocket.

Even though the hollow fibers acted as an effective drug retaining device, they allowed for quick drug evacuation. Chitosan has the capacity to form fibers. Electrospinning is a simple and economic method for producing chitosan fibers with size varying from nanometers to microns for use primarily in musculoskeletal and brain tissue engineering [96]. Moreover, because of the inferior mechanical qualities of this kind of chitosan, several changes were introduced [97]. The synthesis of key characteristics of fiber meshes and chitosan fibers used for biomedical applications such as periodontal bone regeneration has been studied [98]. Tetracycline fiber administration as an adjunct to first line therapy led to significantly decreased Regression of periodontitis when compared to SRP alone [99]. Although studies demonstrate that these fibers are clinically helpful, their true impact in patient care has been challenging to identify due to periodontists’ difficulty with the fiber implantation method. Participants in one study confirmed challenges during fiber replacement and removal. The difficulties of wrapping a fiber into place, holding the implant within the pocket, and then releasing it after 7 to 10 days may limit its general acceptance by patients and periodontists [100].

### 6.6. Sponges

Sponges are the spreading of gas in an integrated matrix (usually air). Sponge is increasingly being employed in the biotechnological areas, notably as frameworks for drug carriers, and matrices for cell development in the bioengineering industry. Sponges derived from polysaccharides such as chitosan, in particular, have been investigated, thanks diminished cytotoxic activity, superior mechanical characteristics, and potential for substance bio sorption [101]. Chitosan sponges were studied in terms of bone healing [102], and for wound dressings of the periodontium in which antimicronials may be administered [103]. To evaluate the efficacy of mixed sponges as prospective wound dressings or matrices for tissue engineering, sponges based on sodium alginate and chitosan were freeze-dried [104]. Lee et al. found that Platelet-derived growth factor-BB(PDGF-BB) -containing chitosan sponge increased bone healing. (PDGF-BB) [105].

### 6.7. Composite

Hurt et al. (2014) described a polymer composite of chitosan, polyvinyl alcohol (PVA), and Ag+ exchanged tobermorite (CPTAg) for periodontal administration as antimicrobial chemotherapy [106]. Chitosan with differing molecular weights was also employed to make chitosan-collagen composites, which were then tested in vivo for their osteoinductive activities [107]. The clinical outcome indicates that both chitosan-collagen composite groups significantly increased new bone growth, with no significant variation between different weight chitosan and absence of autoinduction by the comparator group.

## 7. Bioactive Agents Used for Local Periodontal Regeneration

Chitosan scaffolds have the potential to encapsulate many drugs to be implemented as a local drug delivery system in periodontal tissue regeneration. Table 5 shows examples of chitosan scaffolds loaded with drugs in periodontal regeneration.

### 7.1. NSAIDs

#### 7.1.1. Aspirin

Acetylsalicylic acid (ASA), often referred to as aspirin, can stimulate bone repair. Countless papers have commented on its biological mechanisms, the majority of which are connected to other immunomodulation, such as T cell inhibition, MSC life span extension, and immunomodulation ability augmentation [117]. Chitosan hydrogel encapsulating acetylsalicylic acid (ASA) exhibited sustained release for more than 14 days, encouraging PDLSC proliferation and osteogenic differentiation [118]. Chitosan (CS)/b-sodium glycerophosphate/gelatin hydrogels containing aspirin/erythropoietin (EPO) have been shown to be useful in the treatment of periodontal inflammation and regeneration [119].

#### 7.1.2. Ibuprofen

Ibuprofen was approved as an anti-inflammatory medicine in the United Kingdom in 1967 and in the United States in 1974. It possesses weak but evident anti-inflammatory qualities equivalent to aspirin, milligram for milligram, but with significantly less gastrointestinal irritation [120]. Thermo-responsive in-situ micro gels packed with the antimicrobial agent metronidazole (MTN) and the no steroidal anti-inflammatory drug ibuprofen (IBU) gave good MTN release after 8 h and sustained IBU release for 24–48 h, suggesting that they could be used as periodontitis treatment systems [121].

#### 7.1.3. Meloxicam

Meloxicam is a no steroidal anti-inflammatory drug (NSAID) that prevents cartilage and bone loss [122]. Biocompatible chitosan (CS)/poly (vinyl alcohol) (PVA)/hydroxyapatite (HA) electro spun (e-spun) fibers and films filled with meloxicam were shown to be non-cytotoxic, and cells proliferated well on these synthetic scaffolds. These characteristics, together with our produced compounds’ immunomodulatory potential, hint to their utility in periodontal therapy [123].

### 7.2. Antimicrobials

#### 7.2.1. Clindamycin

Clindamycin is a broad-spectrum bacteriostatic lincosamide used in dental and periodontal therapy. It is a water-soluble medicine having a 2.9-h biological half-life [71,124,125,126,127]. Additional study is needed to produce and evaluate clindamycin phosphate loaded chitosan/alginate polyelectrolyte complex films as a mucoadhesive medication carrier for periodontal treatment [45].

#### 7.2.2. Doxycycline 

Doxycycline is a semi-synthetic antibiotic produced from the species Streptomyces that includes vicinal dials able to bind with divalent ions and inhibiting metabolic activity. Doxycycline inhibits the formation of peptide bonds in bacteria by preventing amino acid addition. [128]. Doxycycline, in addition to its antibacterial effects, inhibits matrix metalloproteinases (MMPs) [129]. The method of activity of doxycycline treatment for rheumatoid arthritis is anti-collagen lytic. This is owing to its metal-binding property.

Doxycycline treatment at a sub-antimicrobial dose has been demonstrated to diminish periodontal disease activity by lowering MMPs and pro-inflammatory mediators [130]. Doxycycline suppresses host-derived MMPs through mechanisms unrelated to its antibacterial effects. It’s both safe and reliable at clinical doses and has been successfully studied in numerous conditions linked with high MMP activity.

MMP activity has been demonstrated to be reduced by doxycycline in arthritis and periodontitis [131], and aortic aneurysms [132]. Adjunctive sub-antimicrobial dosage doxycycline therapy can help enhance clinical characteristics in chronic adult periodontitis by reducing the amount of MMP 8 [130].

Doxycycline-containing chitosan/carboxymethyl chitosan nanoparticles demonstrated excellent tolerability and successfully reduced NLRP3 inflammasome and IL-1 gene and protein expression in human gingival fibroblasts (HGFs). 

#### 7.2.3. Metronidazole

At low doses, metronidazole (MET), a nitro imidazole molecule, has been shown to be bactericidal to most anaerobic bacteria such as bacteroides, fusobacteria, and treponemes.

Another interesting feature is that MET kills most Gram-negative anaerobic bacilli without altering the health-related flora.

Furthermore, numerous investigations have shown that resistance development with anaerobes is uncommon with MET [133].

Chitosan in-situ gel encapsulating levofloxacin and metronidazole was thermo sensitive, mucoadhesive, syringe able, and gradually and consistently delivered medications against a diverse range of microbes [134].

#### 7.2.4. Minocycline

Minocycline is a second-generation tetracycline antibiotic that is semi-synthetic. It is a wide-ranging antibiotic, developed in 1967, that is used in the control and treatment of a wide range infectious and non-infectious illnesses, having anti-infectious activity comparable to other tetracyclines. It possesses anti-infective efficacy against wide range of bacteria, as well as anti-inflammatory, anti-oxidant, anti-apoptotic, and immunomodulatory features. Because it is a highly lipophilic compound that may pass the blood-brain barrier, it is recognized to be the most powerful tetracycline derivative at producing neuroprotective benefits [135]. Moreover, minocycline has pro-anabolic and anti-catabolic effects on periodontal tissues, lowering the levels of bone resorption mediators and increasing bone formation and performance [136].

Minocycline-loaded chitosan nanoparticles (MH-NPs) have the capability to be implemented in the management of periodontal disease by combining intracellular drug targeting with strong anti-inflammatory properties [137].

#### 7.2.5. Moxifloxacin

Moxifloxacin is a 4th fluoroquinolone with enhanced efficacy against gram-positive bacteria as well as unusual illnesses [138].

Cross-linked chitosan films containing moxifloxacin were ductile, had satisfactory mechanical properties, and had excellent physicochemical features.

Even though films release medicine immediately, it was maintained for approximately 15 days, showing that it might be useful in local periodontitis treatment [139]. 

#### 7.2.6. Ornidazole

It is a commercially available synthetic nitro imidazole. The antimicrobial activity is similar to metronidazole; however, it is better tolerated [140]. Injectable chitosan/-glycerophosphate scaffolds containing long-lasting BMP-7 and ornidazole promoted new bone formation by boosting osteoblastic activity and lowering osteoclast activity [141].

#### 7.2.7. Tetracycline

Tetracyclines are antibiotics with a broad spectrum of activity [142]. The cytocompatibility of tetracycline hydrochloride-loaded PVA and chitosan scaffolds was validated, demonstrating that the studied scaffold may be employed as antimicrobial wound healing promoter [143].

#### 7.2.8. Chlorhexidine

The proposed medication was chlorhexidine gluconate (Chx). Because of its action against a wide spectrum of bacteria species, it is commonly used in clinical dental treatment as an antiseptic oral rinse [144]. Chitosan nanoparticles when combined with chlorhexidine resulted in enhanced tissue regeneration after periapical surgery [145]. An injectable chlorhexidine/Cs thermo sensitive hydrogel demonstrated excellent biocompatibility and remarkable antimicrobial activity, making this hydrogel a promising candidate for local periodontal tissue regeneration.

### 7.3. Statins

Statins are powerful cholesterol-lowering medications that act by blocking a crucial component in the cholesterol production pathway. They have made major advancements in cardiovascular disease prevention [146]. Statins have anti-inflammatory and immunomodulatory characteristics because they reduce the stimulation of inflammatory cytokines such as interleukin 1 (IL-1) and interleukin 6 (IL-6), and tumor necrosis factor (TNF-α) [147,148,149,150]. Furthermore, statins can stimulate osteoblast development by boosting bone morphogenetic protein-2 (BMP-2) which is consider to be a stimulant for osteoblast differentiation and bone production [151]. They can also activate the vascular endothelial growth factor (VEGF), which stimulates bone tissue development [152]. Statins have been shown to be effective in increasing bone growth for human periodontal ligament cells due to their anti-inflammatory and anabolic actions on the bone [153].

#### 7.3.1. Atorvastatin

Atorvastatin is a statin drug that is used to lower blood cholesterol levels [154]. In recent times, the various properties of atorvastatin have been tested for their potential applicability in the management of a range of inflammatory and immune-mediated illnesses, particularly periodontitis [155,156,157,158,159]. Numerous trials on the local administration of atorvastatin for periodontitis therapy, particularly in gel systems, have been reported [155]. Nevertheless, atorvastatin has a poor water solubility and so has a low bioavailability. The secretion of pro-inflammatory (IL-1, IL-6 and IL-8) and anti-inflammatory (TGF-1 and TGF-2) cytokines was reported to decrease after administration of atorvastatin chitosan gel, with substantial alveolar bone repair. These data imply that a chitosan-based delivery method for atorvastatin, a statin group medication, is potential for the management of periodontal disease [155].

#### 7.3.2. Lovastatin

Lovastatin is a statin drug that is used to treat and prevent coronary heart disease, hypercholesterolemia, and adolescent individuals with hereditary hypercholesterolemia [160].

Chitosan membrane loaded with epigallocatechin-3-gallate and lovastatin showed strong alkaline phosphatase efficiency and antimicrobial activity against common pathogenic bacteria, resulting in better bone healing. These findings imply that the EGCG14-CS-Lovastatin1 membrane could be employed as a new GTR membrane [161].

### 7.4. Hormones

#### 7.4.1. Thyroxin

Thyroxin is a necessary hormone that performs a variety of physiological functions in the human body. One of them is its ability to induce angiogenesis in a variety of ways [162]. Thyroxin stimulates the production of basic fibroblast growth factor (bFGF) and vascular endothelial growth factor (VEGF) via increasing integrin v3, the two key growth factors required for vasculature development [163]. Thyroid hormones had an effect on cellular metabolic responses as well as cell proliferation [164]. The capacity of chitosan/collagen-based thyroxin-loaded hydrogels to neovascularize suggests that they could be beneficial materials for future tissue engineering applications [162]. 

#### 7.4.2. Dexamethasone

DEX, a corticosteroid, has been found to enhance osteoblast development in vitro and bone tissue formation by activation of osteoblasts [165]. DEX has long been employed as an osteoinductive agent due to its high stabilization and osteogenic action [166]. High DEX levels, on the other hand, would hinder osteoblast formation and cause dangerous side effects [166]. Therefore, its potential for use in bone regeneration is restricted. Thus, extended DEX release from scaffolds is required to maximize efficacy while minimizing deleterious effects on bone regeneration. Porous bio composite scaffolds based on chitosan and dexamethasone, had an osteoinduction capacity on rat bone marrow stem cells, and could be promising local implantable scaffolds for bone regeneration [167]. In the late stages of differentiation, DEX has been found to stimulate bone marrow cell differentiation as well as lead cells toward final maturation [168]. Dexamethasone-infused chitosan-alginate-gelatin matrix was reported to promote proliferation and osteogenic differentiation [167].

#### 7.4.3. Raloxifene

Raloxifene (RLX) is an osteoporosis medicine that is a second-generation selective estrogen receptor modulator (SERM). Raloxifene has an estrogen-like effect on bone and has been shown to promote bone mass density (BMD) and bone health [169]. In the study of Zhang et al. [170]. In vitro testing was carried out utilizing a scaffold made of chitosan, collagen, and -TCP that was loaded with PLGA microspheres containing RLX, with RLX dosages ranging from 0.1 to 10 g. The findings showed that the RAL was gradually released, resulted in superior cell survival at all concentrations, and significantly improved cell proliferation, mineralization capability, and ALP activity. A TiO_2_ nanotube arrays (TNT)/raloxifene (RLX)/layer-by-layer/alendronate (RLX/LBL-Aln) implant in osteoporotic rabbits may efficiently expedite the production of new bone surrounding the implant and increase bone binding [171].

#### 7.4.4. Erythropoietin

Erythropoietin (EPO), a glycoprotein recognized as a key erythropoiesis stimulator, is secreted by kidneys in adults and liver during intra uterine life [172]. Erythropoietin (EPO) is a low-molecular-weight glycoprotein hormone that induces erythropoiesis. RhEPO was released in 1989, and it is now utilized cure anemia caused by renal insufficiency, chemotherapy, bone marrow transplantation, and AIDS [173]. Erythropoietin stimulates the synthesis of anti-oxidative enzymes, inhibits glutamate cytotoxicity, metabolizes free radicals, normalizes cerebral blood flow, affects neurotransmitter release, and promotes neoangiogenesis [174]. Unlike previously-held notions that EPO was only advantageous in the creation of erythropoiesis, Epo has been proven to have pleiotropic effects in a range of cell types, including anti-apoptosis [175,176]. There is rising evidence that EPO has biological roles in tissues other than the hematopoietic system, which has piqued the interest of researchers. EPO is a tissue-protective hormone that promotes wound healing after ischemia-reperfusion, trauma, cytotoxic infections, and tissue/organ inflammation [177]. Topical treatment of recombinant human EPO applied to injuries in diabetic rats and mice promotes wound healing by boosting revascularization, epithelization, and collagen accumulation while reducing the inflammatory reaction and apoptotic cascades [178]. Recent study has revealed that EPO also has a role in bone homeostasis.

EPO may enhance bone formation by increasing the synthesis of vascular endothelial growth factor, one of the most significant growth factors in bone repair and regeneration for stimulating angiogenesis and vascularization [179] and bone morphogenetic protein 2 [180]. EPO also influences bone development via mTOR signaling [181]. According to the results of a study conducted by Li, C et al., EPO enhances osteoblastic activity via EphB4 signaling while increasing the number of ephrinB2-expressing osteoclasts while decreasing their resorptive activity. Bone growth was caused by the combination of bidirectional signals mediated by EPO via ephrinB2/EphB4 signaling [182]. EPO therapy has been demonstrated to improve wound healing by increasing cell proliferation and angiogenesis, which is associated with increased production of VEGF, endothelial nitric oxide synthase (eNOS), and inducible nitric oxide synthase (iNOS) (iNOS) [183,184]. VEGF is an important protein that promotes angiogenesis and osteogenesis during bone repair [185,186]. Recent research has also demonstrated that VEGF therapy enhances the osteogenesis pathway [186]. Similarly, nitric oxide synthases, which are generated by pericytes and chondrocytes within the fracture callus, have been shown to be critical in bone regeneration [187,188]. Furthermore, between the third and fourth weeks following free gingival transplant procedures, topical EPO treatment accelerates palate wound healing [189]. Aslroosta et al. published preliminary studies in 2021 demonstrating that EPO showed potential in the treatment of people with moderate to severe chronic periodontitis [190]. In a study, Wang et al. discovered that Erythropoietin enhances the osteogenesis of periodontal mesenchymal stem cells from healthy and periodontitis sources [191]. In a study led by them, Li et al. revealed that an injectable thermo responsive hydrogel packed with erythropoietin can successfully improve maxillary sinus floor augmentation [192]. Injectable thermosensitive hydrogels containing erythropoietin and aspirin were discovered to induce periodontal regeneration [119].

### 7.5. Hemostatic Drugs

E-aminocaproic acid (EACA) is a synthetic plasmin-plasminogen inhibitor. It is the only effective antifibrinolytic drug on the market in the United States [193]. By modulating fibrin biodegradability, fibrin-Aminocarpoic acid-Chitosan particles can enhance cementogenesis and osteogenesis, implying the possibility of its therapeutic usage to improve periodontal regeneration [116].

## 8. Conclusions

Chitosan has evolved as a polymer with a wide variety of possibilities due to its diversified biological activity, excellent biocompatibility, and complete degradability, as well as its low toxicity. Chitosan contains an uncommon mix of biological activity, mechanical properties, and physical properties. It is a common natural biopolymer having diverse uses in medicine, cosmetics, food processing, and biotechnology. Chitosan applications have been delayed due to concerns about its intractability, low solubility, restricted surface area, and porosity. Even though chitin and chitosan have been named our “last biomass resource” and are expected to provide novel functional polymers, their application has vast unexplored potential. There has been a surge of attention in employing naturally occurring chemicals with distinct antibacterial and hemostatic capabilities in recent times. Exploring the vast range of uses for chitosan and its variants is challenging but rewarding. Truthful achievement of this objective will transform chitosan from a compound with potential into a vital player in several critical applications. 

## Figures and Tables

**Table 1:** Chitin existence in nature.

Phylum	Class
Arthropoda	Crustaceans (e.g., Shripms), Arachnida (e.g., spiders)
Mollusca	Cephalopoda (squid, octopus)
Annelida	Polychaeta (Bristle worms)
Fungi	Ascomycota, Basidiomycota
Protozoa	Rhizopoda (amoeba)

**Table 2:** Chitosan’s benefits and drawbacks as a local medicine delivery vehicle.

Advantages	Disadvantages
Minimal Toxicity	Low mechanical resistance
Improved Biocompatibility	Uncontrolled pore size
Have a mucoadhesive features	Possibility of contraction
Stable	Pure chitosan is difficult to electrospin.
Site-specific drug delivery	Crosslinking can alter the intrinsic characteristics of chitosan.
Increased therapeutic index	Poor solubility except in acidic media.
Repeated, costly, and undesirable dosing is avoided.	The method of manufacturing process must be altered depending on the medicine to be administered.

**Table 3:** Fabrication methods of chitosan products, application, advantages and disadvantages [41].

Fabrication Method	Carrier	Loaded Drug	Form	Application	Advantages	Disadvantages	Reference
**Ionic gelation**	Chitosan/carboxymethyl chitosan nanoparticles	Doxycycline	Nanoparticles	Periodontal implant	-Less toxic -Higher stability -Therapeutic index of the drug is enhanced	-Low Solubility -Mechanical resistance is less	[42]
**Solvent evaporation**	Chitosan	metronidazole	microparticles	Periodontal implant	-Simple -Economic	-Difficulty of controlling the evaporation rate. -Presence of impurities	[43]
**Reverse micellar**	Sulfobutylether β- cyclodextrin/chitosan nanoparticles (SBEβCD/CS NPs)	Asiaticoside (AS)	Gel	Periodontal implant	Ability to produce a small particle size with a narrow size of the distribution	Laborious and time-consuming process and the presence of organic solvent and surfactant	[44]
**Cross-linking**	chitosan/alginate polyelectrolyte complex	clindamycin phosphate	Film	Periodontal Implant	-Soft and flexible networks. -Mucoadhesive	-Uncontrolled dissolution.	[45]
**Self-assembling**	polyelectrolytes carboxymethyl chitosan (CMC) and polylysine (PLL)	Metformin	Scaffold	-Surface coating	-Save energy. -Protection of encapsulated drugs from disrupting forces.	-Sophisticated procedure	[46]
**Sieving**	Chitosan	Triclosan	Nanoparticles	**-Anti-inflammation**	**-easy handling. –low costs, -precise and reproducible**	-Presence of hazardous residues	[47]
**Spray Drying**	Chitosan dispersed in polyvinyl alcohol (PVA)	Doxycycline	Gel	-Scaffold	-Simple -Reproducible	-High cost	[48]
**Freeze-Drying**	β-tricalcium phosphate (β-TCP), chitosan (CTS) and the mesoporous silica (SBA-15)	Metformin	Composite	Scaffold	-prolonged shelf-life. -Improved physical properties	-Inability to preserve bioactivity.	[49]

**Table 4:** Advantages and disadvantages of different formulations of chitosan as local drug delivery system.

Formulation	Advantage	Disadvantage
Gel	Ease of application, sustained drug release, and good patient compliance.	Needs large volume.
Hydrogel	Controlled drug release, and can take the shape of the deformity	lack of mechanical strength.
Micro particles	Ease of application, controlled drug release.	Difficulty in retaining in the application site.
Fibers	Can be used in distant area, such as last tooth in the jaw.	Needs to be removed and shows some inflammation.
Nanoparticles	Precise application.	Lack of stability and sophisticated manufacturing techniques.
Strips and films	Thin, flexible, and minimal discomfort.	Burst drug release.
Scaffolds	Enhanced mechanical properties, and	Difficult processing, and high cost

**Table 5:** Examples of Drug-Loaded chitosan Scaffolds for periodontal tissue regeneration.

Form of Chitosan	Drug	Technique Used	Description	Reference
Microspheres	Tetracycline HCL		Enhancing bioadhesion, controlling medication distribution, and boosting antibacterial properties	[108]
microspheres	Ofloxacin		Controlled release rate is high, and the pharmacokinetic pattern is advancing.	[109]
Microspheres	Doxycycline	Water-in-oil emulsion	Improves cell survival significantly when compared to pure medication, and performs better in vitro	[110]
Micro particles	Natamycin	Emulsion-polymerization	Antibacterial activity with prolonged drug release.	[111]
Films	Ciprofloxacin hydrochloride	Casting/solvent evaporation	Deliver antimicrobial agents into the periodontal pocket.	[112]
Film	Metronidazole benzoate	evaporation	Sustained drug release, the effective local dose was 15-fold less than the systemic dose.	[113]
Inserts	Metronidazole	Casting method	Initial rapid release shown for reducing pathogenic load in periodontitis	[79]
Films	Ornidazole	Casting methods	Drug bioavailability extended up to six days.	[112]
Gels	Metronidazole		Dissolution rate in gingival crevicular fluid was massively increased multiple folds when evaluated in vivo.	[114]
Gels	Ornidazole		Improved bio adhesivity, prolonged pharmacological activity, and good patient compliance	[115]
Nanoparticles	e-aminocaproic acid	Ionic gelation	Increased cementogenesis and osteogenesis	[116]

## Data Availability

Not applicable.
